# Triphasic clinical course of driveline exit-site inflammation managed outpatient via photographic monitoring: An allied health case report of two left ventricular assist device patients

**DOI:** 10.1016/j.jhlto.2026.100676

**Published:** 2026-08-31

**Authors:** Satoshi Taketani, Shunsuke Saito, Ryohei Matsuura, Asami Sueyoshi, Kaori Kubota, Kazuma Handa, Shigeru Miyagawa

**Affiliations:** a InteDix, Inc., Kobe, Japan; b Department of Cardiovascular Surgery, The University of Osaka Graduate School of Medicine, Osaka, Japan; c The University of Osaka Hospital, Osaka, Japan

**Keywords:** left ventricular assist device (LVAD), driveline infection, personal health record, remote monitoring, Bayesian algorithm, mechanical circulatory support

## Abstract

Driveline infections are a leading cause of morbidity in patients with a home left ventricular assist device (LVAD). We report a case of culture-negative, recurrent driveline exit-site inflammation with a triphasic clinical course (91 days, 14 photographs) in VAD018, managed entirely as an outpatient—without antibiotic therapy—through VAD coordinator-led review of weekly self-photographed personal health record (PHR) submissions via the Minatowa platform. A Bayesian deterioration algorithm, applied retrospectively and incorporating photographic wound scores as a likelihood ratio component, identified all 3 clinical peaks (*p*[deterioration] > 0.80) at timepoints corresponding to coordinator-initiated intensification of wound care. For context, VAD034, a patient without PHR monitoring, experienced a 26-day hospitalization for driveline exit-site inflammation of comparable peak severity, also culture-negative, ultimately managed with incision and irrigation. These findings are hypothesis-generating and motivate prospective evaluation of coordinator-led, PHR-based photographic surveillance, planned as the Local LVAD Home Management (LHM) Trial.

## Background

Driveline infections occur in 10–20% of patients with a left ventricular assist device (LVAD), a mechanical ventricular assist device (VAD) implanted to support cardiac output in patients with advanced heart failure, within the first year and represent a primary driver of hospitalization and mortality.^1^, ^2^, ^3^ Current surveillance relies predominantly on scheduled monthly outpatient visits, limiting the ability to detect early wound changes between appointments. Digital health platforms enabling patient-submitted photographs may bridge this surveillance gap, yet longitudinal photographic evidence in home LVAD management remains scarce.^3^

The Minatowa LVAD-PHR platform allows patients to submit weekly exit-site photographs alongside daily vital sign recordings and free-text communication with the multidisciplinary team. A Bayesian deterioration probability algorithm—incorporating wound photographs as a likelihood ratio component—provides a decision-support risk stratification output. While physician-led telemonitoring programs for heart failure have demonstrated benefit in randomized trials,^3^, ^4^ the specific contribution of allied health professionals—particularly VAD coordinators—in digital photographic surveillance of driveline infections has not been well characterized. Here we present a case report in which a VAD coordinator-led photographic review process was the primary real-time clinical mechanism guiding outpatient management, with the Bayesian algorithm applied retrospectively as a decision-support adjunct for proof-of-concept validation.

VAD coordinators—typically advanced practice providers (APPs)—perform 3 core clinical competencies central to home LVAD management: (1) *clinical assessment and surveillance*, encompassing evaluation of the driveline exit site, device parameters, and haemodynamic status between scheduled physician visits; (2) *patient and caregiver education and self-management support*, including instruction in exit-site wound care technique, dressing protocols, and the recognition of early warning signs; and (3) *multidisciplinary care coordination*, serving as the primary interface among the cardiovascular surgical team, home-care physicians, patients, and caregivers to facilitate timely clinical decision-making. In the present case, all 3 competencies were operationalized through the PHR platform: weekly photographic submissions enabled continuous wound surveillance (competency 1), the patient performed coordinator-directed wound care at home (competency 2), and the APP coordinated with the home-care physician at each of the 3 intensification timepoints (competency 3). This integrated coordinator-led model, rather than algorithmic automation, constitutes the primary clinical mechanism described in this report.

## Methods

**Figure 1:**
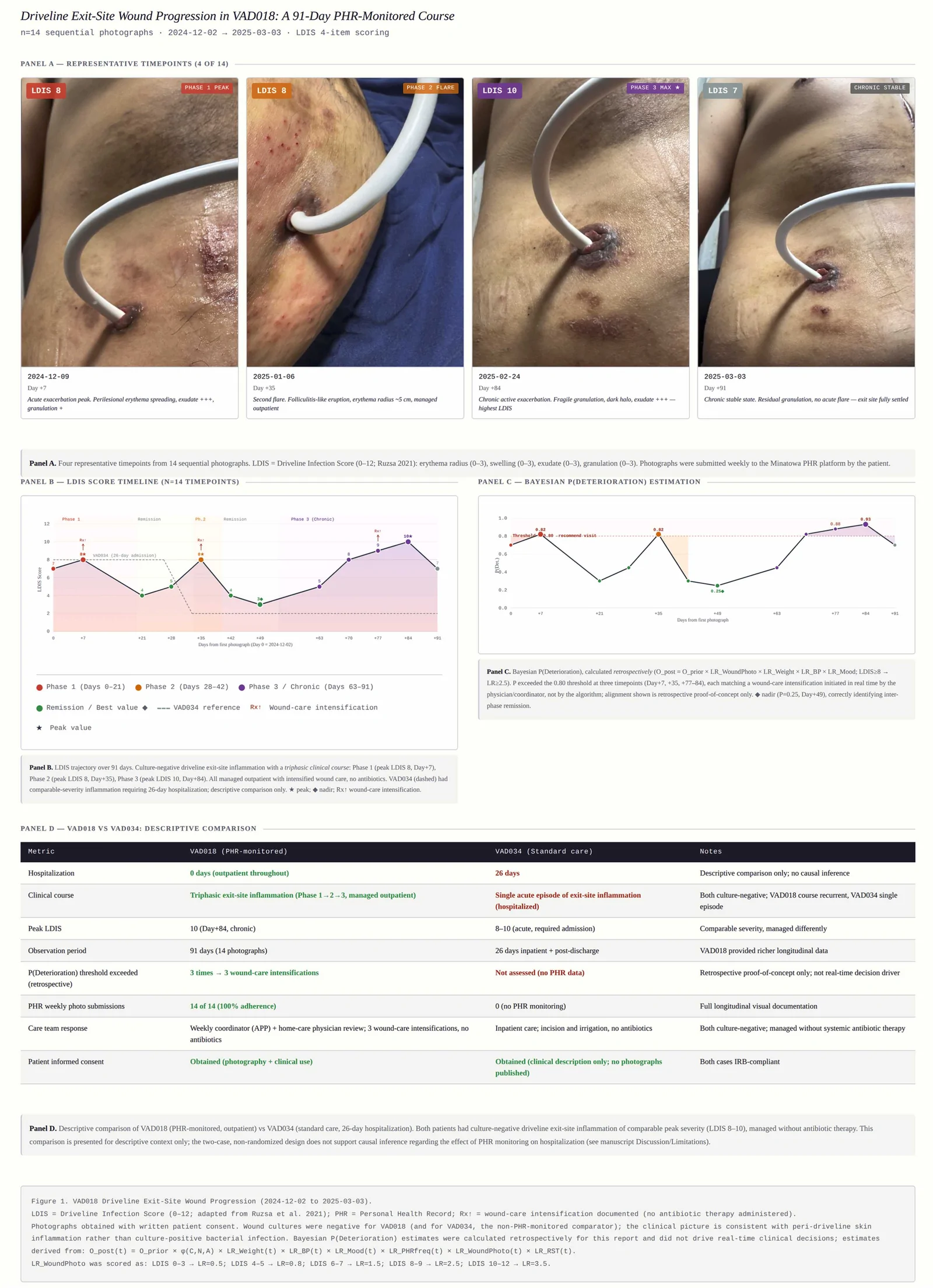
VAD018 driveline wound progression (2024–12-02–2025-03–03; *n* = 14 photographs). Panel A: Representative photographs at 4 timepoints with LDIS overlays: Phase 1 peak (Dec 9, LDIS = 8); Phase 2 flare (Jan 6, LDIS = 8); Phase 3 maximum (Feb 24, LDIS = 10); Chronic stable (Mar 3, LDIS = 7). Patient written consent obtained for all photographs. Panel B: LDIS score trajectory across 12 time-points (solid line) with VAD034 reference (dashed line; 26-day hospitalization). Phase bands: Phase 1 (red, Days 0–21), Phase 2 (orange, Days 28–42), Phase 3/chronic (purple, Days 63–91). Rx↑ = documented outpatient intervention; ★ = peak; ◆ = nadir (LDIS 3, Day +49). Panel C: Estimated Bayesian P (deterioration). Dashed red line: visit-recommendation threshold (*p* = 0.80). Threshold exceeded at Day +7, +35, and +77–84, corresponding to documented interventions. Panel D: Comparative summary table, VAD018 vs VAD034 (8 metrics).; PHR, Personal Health Record.

This retrospective case report, with a second patient (VAD034) presented for contextual comparison, derives from the Minatowa LVAD-PHR cohort (*n* = 42; Osaka University IRB approval no. 24167 (T1)). During the observation period, 5 of the 42 cohort patients experienced a documented driveline exit-site complication; no other cohort patients developed a driveline complication. VAD018 and VAD034 are therefore reported as 2 of the complete set of 5 driveline complication cases in this cohort, rather than as a subset selected from a larger pool of complication cases. VAD018 was the only patient among the 5 with continuous weekly PHR photographic documentation throughout the complication episode; the remaining 4 patients, including VAD034, did not have PHR photographic monitoring and were each managed via standard outpatient clinical assessment, culminating in hospitalization for incision and irrigation. VAD018 and VAD034 are presented in case-level detail for comparison given their comparable peak wound severity (LDIS 8–10); the other 3 non-photographed, hospitalized patients are characterized only in aggregate, as case-level data were not collected for this retrospective report (see Limitations). Written informed consent for participation in the Minatowa LVAD-PHR cohort was obtained from all 42 patients under the parent IRB protocol. In addition, written informed consent for publication of this case report was obtained from VAD018 and VAD034; identifiable clinical photographs (Figure 1) are presented for VAD018 only, with consent for photographic publication obtained from VAD018. Clinical information regarding VAD034 is presented in de-identified, non-photographic form. Sex and gender: both patients were male; sex-based subgroup analysis was not feasible in a 2-patient report (SAGER guideline compliance noted in Limitations).

VAD018 submitted 14 weekly self-photographs of the driveline exit site (December 2, 2024–March 3, 2025) via the Minatowa platform bulletin board using a standard smartphone. Prior to discharge, all Minatowa cohort patients receive standardized driveline exit-site care education and ongoing wound management in accordance with the device- and wound-care recommendations of the JCS/JHFS 2021 Guideline Focused Update on Diagnosis and Treatment of Acute and Chronic Heart Failure.^5^ For VAD018, dressing changes were performed by the patient. Images were reviewed as part of routine weekly PHR check-ins, generally within 1–2 business days of submission; exact turnaround times were not systematically logged for this retrospective report, by a VAD coordinator (in the Minatowa cohort, VAD coordinators are advanced practice providers, APPs; the coordinator reviewing VAD018’s images was an APP, working jointly with the patient’s home-care physician) and a cardiovascular surgeon. VAD034 received standard care without PHR monitoring. Driveline care education and ongoing wound management for VAD034 followed the same JCS-guideline-based institutional protocol as VAD018.

All photographs were scored retrospectively using the 4-item Driveline Infection Score (LDIS; 0–12): perilesional erythema radius (0–3), swelling (0–3), exudate (0–3), and granulation tissue (0–3).^6^ The LDIS adapts the wound-assessment domains of the DESTINE staging framework^6^ (perilesional erythema, swelling, exudate, granulation tissue) for retrospective quantitative scoring of patient-submitted photographs, assigning 0–3 points to each domain (total 0–12) rather than applying DESTINE's ordinal staging categories; this adaptation was necessitated by the requirement to assess features reliably from a single 2-dimensional image (e.g., erythema radius rather than palpable induration). A formal item-by-item comparison with the DESTINE framework was beyond the scope of this case report; we recommend that future validation work, including the planned LHM Trial, incorporate this systematic comparison together with a reference-image scoring rubric. Photographs were scored by a VAD coordinator and a cardiovascular surgeon, both familiar with the DESTINE^6^ wound-assessment scoring framework through clinical experience in the Minatowa LVAD-PHR program; a formal written training protocol or reference-image rubric specific to this report was not developed, and formal inter-rater reliability (e.g., weighted kappa) was not assessed (see Limitations). The Heart Note OS Bayesian algorithm was applied retrospectively: *p*(deterioration) = O_post/(1 + O_post), where O_post incorporates likelihood ratios from vital signs, PHR adherence, and LDIS-derived wound scores (LDIS ≥8: LR = 2.5; LDIS 10–12: LR = 3.5). Visit-recommendation threshold: *p* > 0.80. Importantly, this algorithm and threshold were applied retrospectively for this report only; the 3 clinical interventions described below were initiated in real time by the VAD coordinator and cardiovascular surgeon based on direct visual and clinical assessment of the weekly photographs, not by the algorithm. The retrospective alignment between algorithm output and these coordinator-initiated interventions is therefore presented as proof-of-concept validation of the algorithm against real-world clinical decision-making, rather than evidence that the algorithm itself drove management.

Patient characteristics: VAD018 was a 44-year-old man with dilated cardiomyopathy supported by a HeartMate 3 for 28 months at the time of the index episode (18 months of which included PHR use), with otherwise good driveline exit-site care and no prior hospitalization for driveline-related issues. VAD034 was a 55-year-old man supported by a HeartMate 3 device. VAD034 presented with driveline exit-site pain, bleeding, and granulation tissue formation; because bleeding from the granulation tissue and exit-site pain did not improve with outpatient management, the exit site was incised and irrigated. Exudate and surrounding erythema subsequently improved and the patient was discharged. As with VAD018, wound cultures for VAD034 were negative, and the clinical picture was consistent with peri-driveline skin inflammation rather than a culture-positive bacterial infection (see Discussion).

## Results

VAD018 demonstrated a driveline infection with a previously unreported triphasic clinical course (Figure 1B; see Discussion for definition of “triphasic” as used here). Phase 1 (Days 0–21): acute exacerbation, peak LDIS 8 (Day +7), resolved with intensified wound care (cleansing and dressing changes) to LDIS 4 by Day +21. Phase 2 (Days 28–42): recurrence with folliculitis-like eruption, peak LDIS 8 (Day +35), reaching nadir LDIS 3 (Day +49) within 7 days of intervention. Phase 3 (Days 63–91): chronic progression with maximum LDIS 10 (Day +84); despite the highest wound score of the entire course, no hospitalization was required. Total hospitalization days: 0.

The 3 coordinator-initiated interventions at Day +7, Day +35, and Day +77–84 each consisted of escalated driveline exit-site wound care—increased frequency of wound cleansing and gauze dressing changes—directed by the home-care physician and the VAD coordinator (an advanced practice provider, APP) and performed by the patient. No systemic or topical antibiotic therapy was administered at any of the 3 timepoints; wound condition (erythema, exudate) improved with intensified wound care alone at each phase. Wound cultures obtained for VAD018 at the time of peak severity were negative, consistent with peri-driveline skin inflammation rather than a culture-positive bacterial infection.

Bayesian retrospective analysis demonstrated P(deterioration) exceeding the 0.80 threshold at 3 timepoints—Day +7 (*p* = 0.82), Day +35 (*p* = 0.82), and Days +77–84 (*p* = 0.88–0.93)—each corresponding to a documented outpatient intervention (Figure 1C). The nadir *p* of 0.25 at Day +49 correctly identified the inter-phase remission. PHR photograph adherence was 100% (14/14 submissions).

VAD034 required 26-day hospitalization for a single episode of culture-negative driveline exit-site inflammation of comparable peak severity (LDIS 8–10), presenting with exit-site pain, bleeding, and granulation tissue; the exit site was incised and irrigated, after which erythema and exudate improved without PHR monitoring. Key comparative metrics are summarized in Figure 1D. Beyond VAD034, 3 additional cohort patients without PHR photographic monitoring experienced a driveline exit-site complication during the observation period, each requiring hospitalization for incision and irrigation; case-level data for these 3 patients were not collected for this retrospective report. Together with VAD034, all 4 cohort patients without photographic monitoring who experienced a driveline complication were hospitalized, whereas the sole photographed patient (VAD018) was managed entirely as an outpatient.

## Discussion

The principal finding is a driveline exit-site infection with a triphasic clinical course—3 discrete peaks of LDIS ≥8 over 91 days, each separated by partial resolution to LDIS ≤4—managed entirely as an outpatient through coordinator-led PHR-based photographic surveillance. We use “triphasic” descriptively to denote 3 temporally distinct clinical peaks within a single observation period. Wound cultures obtained for VAD018 were negative, and clinical assessment at each peak was consistent with peri-driveline skin inflammation (dermatitis/folliculitis-type reaction at the exit site) rather than a culture-positive bacterial infection with bacteremia. We therefore interpret the triphasic course as recurrent exacerbations of a single underlying inflammatory process at the driveline exit site, rather than 3 discrete reinfection events with different organisms; this interpretation is consistent with the culture-negative findings in both VAD018 and VAD034 (see Methods and Limitations). This natural history has not been previously described in the LVAD literature, which largely characterizes single-episode acute infections managed during hospitalization.^1^, ^2^ Its identification was only possible because weekly photographs provided continuous wound documentation unavailable with monthly visit schedules.

The retrospective alignment between Bayesian algorithm outputs and the coordinator-initiated clinical interventions provides proof-of-concept validation of the algorithm as a future decision-support tool, rather than evidence of its real-time clinical impact in this report. All 3 *p* > 0.80 threshold crossings corresponded to documented management escalations, and the nadir *p* of 0.25 correctly identified the inter-phase remission. Prospective validation is being designed as the Local LVAD Home Management (LHM) Trial, (RCT; 30-day run-in + non-inferiority design; primary endpoint: rehospitalization-free survival; Group A: full PHR + monthly specialist visit; Group B: full PHR + quarterly specialist visit + monthly primary care).

The comparison with VAD034 is presented for descriptive context only and does not permit causal inference. Both patients experienced peak infection severity of LDIS 8–10; VAD034, who did not have PHR photographic monitoring, required 26-day hospitalization (see Results for hospitalization detail). This pattern was consistent across the cohort: all 4 patients without photographic PHR monitoring who experienced a driveline complication during the observation period were hospitalized, whereas the only patient with continuous photographic monitoring (VAD018) avoided hospitalization entirely; given the small numbers involved (*n* = 5), this complete-cohort pattern remains descriptive and hypothesis-generating rather than causal. One hypothesis consistent with this observation is that weekly photographic surveillance and coordinator-led review may facilitate earlier outpatient intervention before infection severity necessitates inpatient management; this hypothesis is broadly consistent with, though not directly tested by, digital health literature describing reduced acute decompensation through remote monitoring in heart failure populations.^3^, ^5^ We note that the telemonitoring interventions described in references^3^, ^5^ involved care-team structures that may differ from the VAD coordinator-led model described here (see also Limitations), and any comparison should be made cautiously pending prospective, adequately powered evaluation.

This report has several important limitations. First, the 2-patient case-level design precludes statistical inference; no causal relationship between PHR monitoring, coordinator-led review, and hospitalization avoidance can be established, and the findings should be regarded as descriptive and hypothesis-generating only. Second, although wound cultures were obtained and were negative for both VAD018 and VAD034, blood cultures and detailed microbiological characterization (e.g., quantitative wound microbiome sampling) were not performed; while the negative wound cultures support our interpretation of a culture-negative inflammatory process rather than a culture-positive bacterial infection, we cannot fully exclude a low-burden or fastidious organism not detected by standard culture (see Discussion). Third, the 4-item LDIS used here was adapted for retrospective photographic scoring but has not undergone formal validation or inter-rater reliability testing (e.g., weighted kappa) in this report; prospective validation is planned as part of the LHM Trial. Fourth, the Bayesian deterioration algorithm was applied retrospectively and did not influence the real-time clinical decisions described; its retrospective alignment with coordinator-initiated interventions constitutes proof-of-concept only. Fifth, VAD018 and VAD034, together with 3 additional non-photographed and hospitalized patients, represent the complete set of 5 driveline complication cases in this 42-patient cohort during the observation period (see Methods); case-level data for the 3 additional patients were not collected for this retrospective report, and we cannot exclude residual confounding from unmeasured factors among these 5 patients. Sixth, both patients presented in case-level detail were male, precluding sex-based subgroup analysis (SAGER guideline compliance noted). Prospective, randomized evidence from the LHM Trial, including planned inter-rater reliability assessment of LDIS and systematic microbiological sampling, is required to address these limitations.

## Conclusion

In this case report, VAD coordinator-led review of weekly PHR-based photographs was associated with identification of a driveline infection with a previously undescribed triphasic clinical course and with outpatient management of all 3 clinical peaks without hospitalization, in contrast to a 26-day hospitalization in a comparator case without PHR monitoring. Bayesian algorithm outputs, applied retrospectively, aligned with all 3 coordinator-initiated intervention timepoints, providing proof-of-concept support for the algorithm as a future decision-support tool. These findings are hypothesis-generating and support the feasibility of structured, coordinator-led digital wound photography in home LVAD management, motivating prospective evaluation in the planned LHM Trial.

## Financial support

The Heart Note OS platform was developed by InteDix, Inc. (Satoshi Taketani, representative director). The platform was used to collect and store the PHR photographs analyzed in this report. The retrospective case report design, data analysis, interpretation, and decision to submit for publication were carried out independently of InteDix, Inc., by the academic authors. No commercial entity had any role in the review or approval of the manuscript prior to submission.

### Declaration of generative AI and AI-assisted technologies in the writing process

The authors used AI-assisted tools (Claude, Anthropic) to support manuscript drafting, language editing, and figure generation. All intellectual content, clinical interpretation, and scientific conclusions were the sole responsibility of the authors. AI tools were not used as a substitute for human critical thinking or expertise.

## Declaration of Competing Interest

Dr. Taketani is the representative director of InteDix, Inc., the company that developed and operates the Heart Note OS PHR platform, including the Minatowa LVAD-PHR module used in this study. He holds 2 patents related to PHR-based predictive algorithms (JP52928, JP56017). These interests are directly relevant to the technology described in this manuscript. All other authors declare no relevant financial interests.
